# Appropriate seeding rate facilitates the simultaneous enhancement of population yield and lodging resistance in direct-seeded rice

**DOI:** 10.3389/fpls.2025.1622993

**Published:** 2025-08-08

**Authors:** Dongsheng Gai, Yuxin Wang, Haipeng Li, Boting Shi, Yong Liu, Qiang Zhang, Yanqiu Geng, Dongming Ji, Liying Guo, Xiwen Shao

**Affiliations:** ^1^ Agronomy College, Jilin Agricultural University, Changchun, China; ^2^ Siping General Station of Agricultural Technology Extension, Siping, China

**Keywords:** seeding rate, lodging resistance, root system development, main stem and tiller growth, direct-seeded rice

## Abstract

**Introduction:**

Direct-seeded rice is characterized by simplicity, efficiency, and environmental friendliness, with its planting area progressively expanding. However, inappropriate seeding rates can result in issues such as lodging and reduced productive tillers, thereby constraining yield potential. Consequently, this study investigated the response mechanisms of tillering, the heterogeneity between main stems and tillers, and the susceptibility to lodging under varying seeding rates in direct-seeded rice. The aim was to identify an appropriate seeding rate that maximizes yield while mitigating lodging risks, thus providing a theoretical foundation for high-yield cultivation practices.

**Methods:**

Using Jiyujing as the experimental material, a single-factor experimental design was employed, with seeding rates set at 45 kg ha^-1^ (S45), 75 kg ha^-1^ (S75), 105 kg ha^-1^ (S105), and 135 kg ha^-1^ (S135).

**Results:**

Results indicated that the low seeding rate S45 enhanced tillering capacity and productive tillers but was constrained by an insufficient number of effective panicles, limiting overall yield. Conversely, the high seeding rate S135 increased the number of effective panicles but intensified intra-population competition, which hindered individual development, decreased spikelets per panicle, seed setting rate, thousand-grain weight, and lodging resistance, ultimately restricting yield. The intermediate seeding rate S105 achieved the highest yield by balancing population dynamics with individual growth and optimizing the synergy between effective panicle numbers and per-plant productivity. Furthermore, the high seeding rate S135 reduced the diameter, wall thickness, fullness, and physical-chemical component content of basal second internodes, elevating lodging risks. S135 also diminished the average diameter, volume, and surface area of the root system, exacerbating root lodging and yield losses.

**Discussion:**

Therefore, the seeding rate S105 represents the optimal choice for simultaneously enhancing high yield and lodging resistance in direct-seeded rice, offering a theoretical basis for rational plant density management in direct-seeded rice cultivation.

## 1 Introduction

Rice, as a critical grain crop in China, plays a vital role in ensuring national food security (Chen et al., 2021). Rice cultivation primarily involves two methods: transplanting and direct seeding. Direct seeding is a straightforward, efficient, and environmentally sustainable approach to rice farming, which reduces water usage, labor requirements, methane emissions, while enhancing resource utilization efficiency and system productivity (Liu et al., 2015; Tao et al., 2016; Mishra et al., 2017; Jin et al., 2024). Its planting area has been expanding annually. However, in the actual production of direct-seeded rice, factors such as suboptimal seed quality, inadequate cultivation management practices, and adverse climatic conditions often lead to low germination rates and seedling survival rates, thereby reducing the yield of direct-seeded rice (Zhang et al., 2022; Tang et al., 2025). To address this challenge, farmers frequently increase the seeding rate to ensure an adequate number of seedlings, thus boosting the yield of direct-seeded rice; however, this practice also elevates the risk of lodging. The trade-off between achieving high yields and mitigating lodging risks remains a significant issue. Balancing high yield with reduced lodging risk is essential for safeguarding China’s national food security (Shah et al., 2017; Liao et al., 2023).

Seeding rate is a critical factor influencing the establishment of crop population structure (Geleta et al., 2018). A lower seeding rate enhances the tillering capacity of individual plants, yet it leads to a reduction in the total number of panicles; while this approach alleviates intra-population competition and improves resource utilization efficiency per plant, the smaller population size makes it challenging to achieve high yields; conversely, a higher seeding rate exacerbates the competition between individuals and the overall population, diminishing the material production capacity and hindering the formation of an optimal population structure, ultimately resulting in reduced yields; additionally, excessive seeding rates can cause thinner stems, weaker bending resistance, and an increased risk of lodging (Qiu et al., 2016; Meulen and Chauhan, 2017; Matsuyama and Ookawa, 2019; Ahmad et al., 2021; Holman et al., 2021; Khan et al., 2025). Therefore, further investigation into the appropriate seeding rate for direct-seeded rice is essential to balance yield potential with lodging resistance.

Lodging occurs when the gravitational moment acting on the upper part of the stem exceeds the supporting moment due to a combination of internal and external factors, causing the plant to irreversibly shift from its upright position (Mulder, 1954; Jedel and Helm, 1991). This phenomenon typically occurs during the grain-filling stage, when nutrients stored in the stem-sheath are mobilized to the grains, thereby reducing the stem’s fullness, support capacity, and mechanical strength; simultaneously, the weight of the panicle increases as a result of grain filling, rendering rice plants more susceptible to lodging (Mulder, 1954; Feng et al., 2023). The occurrence of lodging disrupts the morphological structure of rice plants, diminishes their photosynthetic capacity, interferes with the grain-filling process, and leads to reductions in both grain yield and quality, as well as in mechanical harvesting efficiency (Setter et al., 1997; Kashiwagi and Ishimaru, 2004; Yadav et al., 2017). Root lodging and stem lodging represent the two primary types of lodging in rice plants, with stem lodging being the most prevalent type in rice production; consequently, research has predominantly focused on stem lodging (Tian et al., 2018; Luo et al., 2023; Rehman et al., 2024; Zhu et al., 2024). However, with the expansion of direct-seeded rice planting areas and the shallower root distribution characteristic of direct-seeded rice, the likelihood of root lodging has increased. Therefore, investigations into lodging in direct-seeded rice should encompass both stem lodging and root lodging.

Previous studies have predominantly focused on the impacts of chemical control measures, fertilizer application, and variations in transplanting density on the population structure and stem lodging resistance of rice (Hong et al., 2022; Zhu et al., 2022; Deng et al., 2023; Xu et al., 2024; Hu et al., 2025; Ma et al., 2025). However, systematic investigations into the effects of different seeding rates on the tillering, the heterogeneity between main stems and tillers, and the lodging resistance of stems and roots remain limited in direct-seeded rice. Therefore, this study employed Jiyujing as the experimental material and established four seeding rates (45 kg ha^-1^, 75 kg ha^-1^, 105 kg ha^-1^, and 135 kg ha^-1^) to systematically elucidate the influence of varying seeding rates on the tillering, the heterogeneity between main stems and tillers, and the response mechanisms related to lodging resistance in direct-seeded rice. The objective was to identify an appropriate seeding rate that could achieve high yields while mitigating the risk of lodging, thereby providing a theoretical foundation for optimizing high-yield cultivation practices in direct-seeded rice.

## 2 Materials and methods

### 2.1 Experimental site

The experiment was conducted at the National Crop Variety Approval Characteristic Identification Station of Jilin Agricultural University in Changchun City, Jilin Province. The soil properties of the experimental field were as follows: organic matter content of 17.13 g kg^-1^, available nitrogen of 46.57 mg kg^-1^, available phosphorus of 10.53 mg kg^-1^, available potassium of 121.80 mg kg^-1^, and pH of 6.1.

### 2.2 Experimental design

The experiment utilized Jiyujing (Jiyujing was developed through a breeding programis at Jilin Academy of Agricultural Sciences. It was created using Hui73 as the female parent and Qiuguang as the male parent. It is mainly used for rice production in the Jilin Province) as the test material and adopted a single-factor randomized block design. Four seeding rates were established: 45 kg ha^-1^, 75 kg ha^-1^, 105 kg ha^-1^, and 135 kg ha^-1^, with three replicates for each treatment. Each plot had an area of 30 m^2^. Rice was directly seeded, and the field was fully prepared under dry-land conditions. The soil underwent deep plowing, compaction, and leveling prior to seeding. Manual hole seeding was performed in early May with a row spacing of 25 cm and a hole spacing of 13.3 cm. Fertilizer application rates were as follows: 150 kg ha^-1^ of pure nitrogen applied in three installments according to the ratio of base fertilizer:tillering fertilizer:panicle fertilizer = 4:3:3; 50 kg ha^-1^ of pure phosphorus applied as a single base fertilizer application; and 75 kg ha^-1^ of pure potassium applied in two installments as base fertilizer and panicle fertilizer at a ratio of 1:1. The nitrogen fertilizer used was urea (N 46%), the phosphorus fertilizer was superphosphate (P_2_O_5_12%), and the potassium fertilizer was potassium sulfate (K_2_O 50%). Water management primarily relied on rain-fed irrigation before the four-leaf stage, during which soil moisture status was monitored and moderate irrigation was conducted when the soil became excessively dry. From the four-leaf stage to one week before harvest, the field was maintained under flooded conditions with a water layer of 3–5 cm. Pest, disease, and weed control were strictly managed throughout the growth period.

### 2.3 Measured items and methods

#### 2.3.1 Tillering dynamics

In 2023, 20 consecutive holes of plants with relatively uniform growth were selected at the four-leaf stage. The occurrence of main stems and tillers in each hole was recorded using the tagging tracking method.

#### 2.3.2 Leaf area and chlorophyll content

In 2023, 10 representative plants were selected at the full heading stage. The length and width of the main stem and tiller leaves were measured to calculate the leaf area of the main stem and tillers. Flag leaves of the main stem and tillers were collected, and chlorophyll content was determined using the acetone-ethanol mixed extraction method.

#### 2.3.3 Spikelets differentiation and degeneration

In 2023, 20 representative plants were selected at the heading stage. Each spike of the main stem and tillers was excised 2 cm below the node and stored at -20°C. The number of differentiated, degenerated, and surviving spikelets was subsequently investigated.

#### 2.3.4 Resistance to lodging measurement

In 2023 and 2024, 10 representative plants were selected 25 days after full heading. Whole plants were harvested and placed in buckets to maintain a non-water-loss condition. Measurements included plant height, center of gravity height, diameter of base second internode, stem fullness of base second internode and wall thickness of base second internode. Bending resistance of base second internode was measured using a stem strength tester (model YYD-1, Top Instrument Co., Ltd., Hangzhou). The bending moment, basal break moment, and lodging index were calculated using the formula (Taiichiro and Kuni, 2008). Samples of base second internode were ground through a 100-mesh sieve for determination of starch and soluble sugar content following the method of Cock and Yoshida (1972). Cellulose and lignin content were determined following the methods of Morrison and Hatfield et al (Morrison, 1972; Hatfield et al., 1999).

#### 2.3.5 Root morphological indicators and root lodging index

Root morphological indicators were assessed using the same samples as those used for stem measurements. Root systems were scanned using an Epson Perfection V800 Photo scanner and analyzed quantitatively with the WinRHIZO root analysis system to obtain root length, root surface area, root volume, and average root diameter. The root lodging index was calculated as the ratio of the product of plant height and fresh weight of a single stem of direct-seeded rice to the product of root dry weight and stem breaking force (Yang, 2007).

#### 2.3.6 Measurement of yield and yield components

In 2023 and 2024, nine representative plants were selected from each plot based on the average number of tillers per plot for investigation of the yield components, and yield was calculated. In 2023, Main stems and tillers were examined separately.

### 2.4 Data analysis

Data statistical analysis was performed using SPSS 25.0 software. One-way analysis (ANOVA) of variance was employed to analyze and assess the significant differences (*P* < 0.05) in the tillering, leaf area and chlorophyll content, spikelets differentiation and degeneration of direct-seeded rice under different seeding rates. Two-way ANOVA was utilized to evaluate the significance (*P* < 0.05) of the interaction between different years and seeding rates on the resistance to lodging, root morphological indicators and root lodging index, and the yield of direct-seeded rice. Pearson correlation analysis was conducted for correlation analysis. Grey relational analysis was conducted using SPSSAU, and graphs were generated using Origin 2024.

## 3 Results and analysis

### 3.1 Effects of different seeding rates on tillering in direct-seeded rice

**Figure 1:**
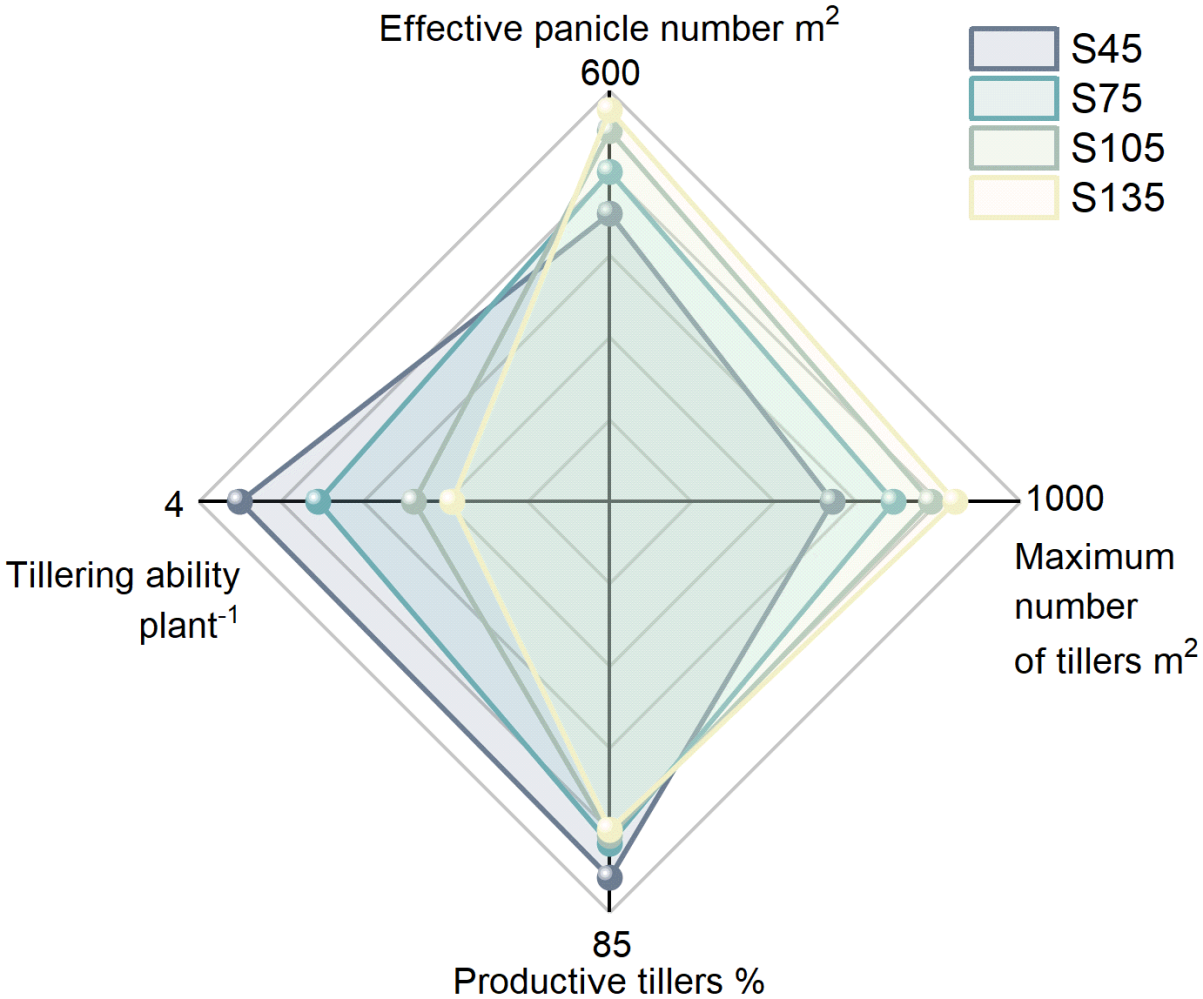
Effects of different seeding rates on tillering in direct-seeded rice in 2023 year. S45: 45 kg hm^-2^, S75: 75 kg hm^-2^, S105: 105 kg hm^-2^, S135: 135 kg hm^-2^.

As the seeding rate increased from S45 to S135, both the effective panicle number and the maximum tiller number in direct-seeded rice gradually increased, reaching their highest values at S135. Conversely, the productive tillers and the tillering ability per plant progressively decreased, attaining their lowest levels at S135 (**Figure 1**).

### 3.2 Effects of different seeding rates on leaf area and chlorophyll content in direct-seeded rice

**Figure 2:**
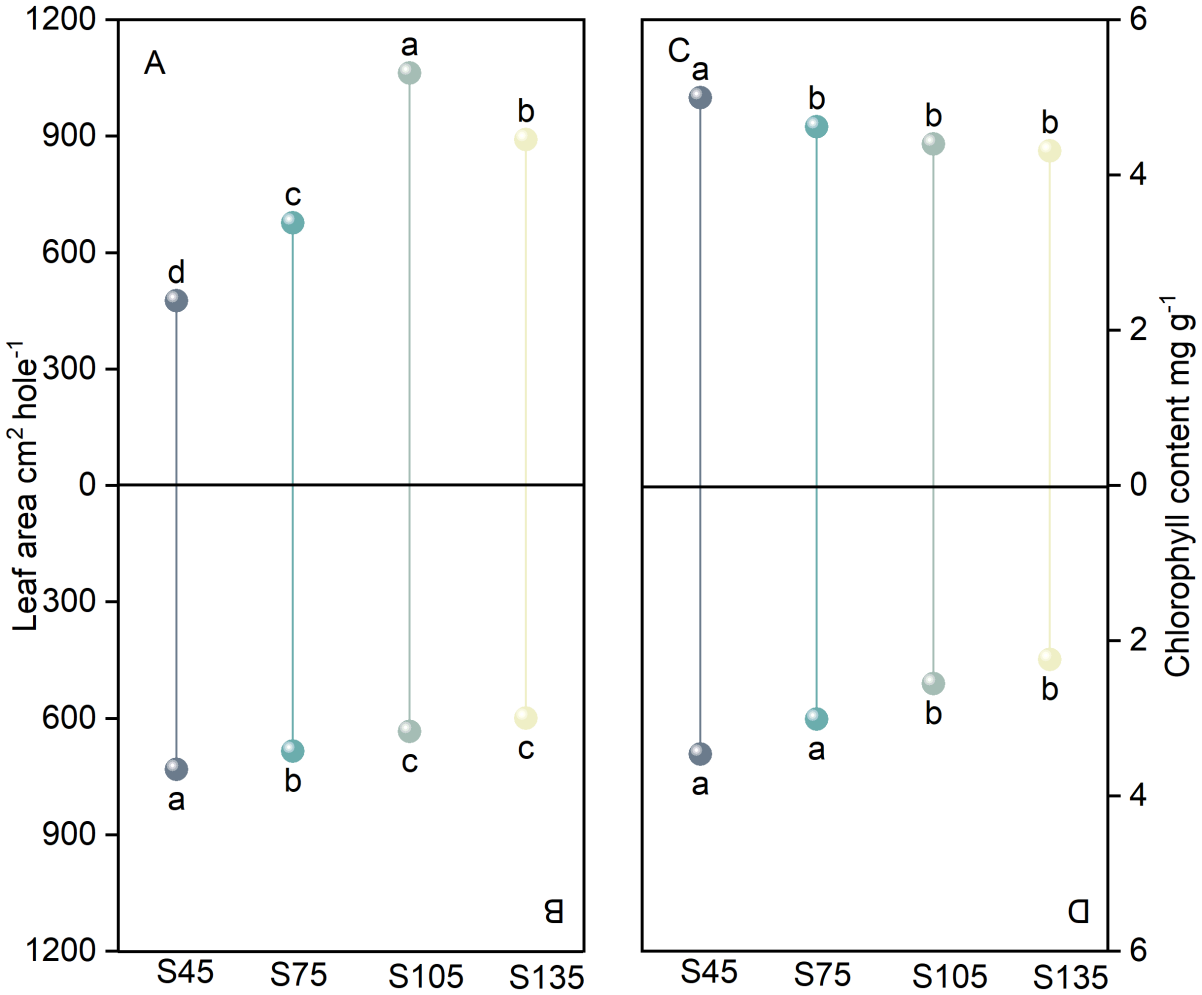
Effects of different seeding rates on leaf area and chlorophyll content in direct-seeded rice in 2023 year. S45: 45 kg hm^-2^; S75: 75 kg hm^-2^; S105: 105 kg hm^-2^; S135: 135 kg hm^-2^. **(A, C)** main stem; **(B, D)** tiller. Different letters indicate significant differences between treatments (*P* < 0.05).

As the seeding rate increased from S45 to S135, the leaf area of the main stem in direct-seeded rice initially increased and then decreased, reaching its maximum at S105 (**Figure 2A**). In contrast, the leaf area of tillers exhibited a gradual decrease, with the highest value observed at S45 (**Figure 2B**). At S45, the leaf area of the main stem was slightly smaller than that of tillers; however, as the seeding rate increased, the leaf area of the main stem progressively expanded, resulting in an increasing disparity between the main stem and tillers (**Figures 2A, B**). Furthermore, as the seeding rate increased from S45 to S135, the chlorophyll content of both the main stem and tillers followed the trend of S45 > S75 > S105 > S135. Under the same seeding rate, the chlorophyll content of the main stem consistently exceeded that of tillers (**Figures 2C, D**).

### 3.3 Effects of different seeding rates on the differentiation and degeneration of primary and secondary branch spikelets in direct-seeded rice

**Figure 3:**
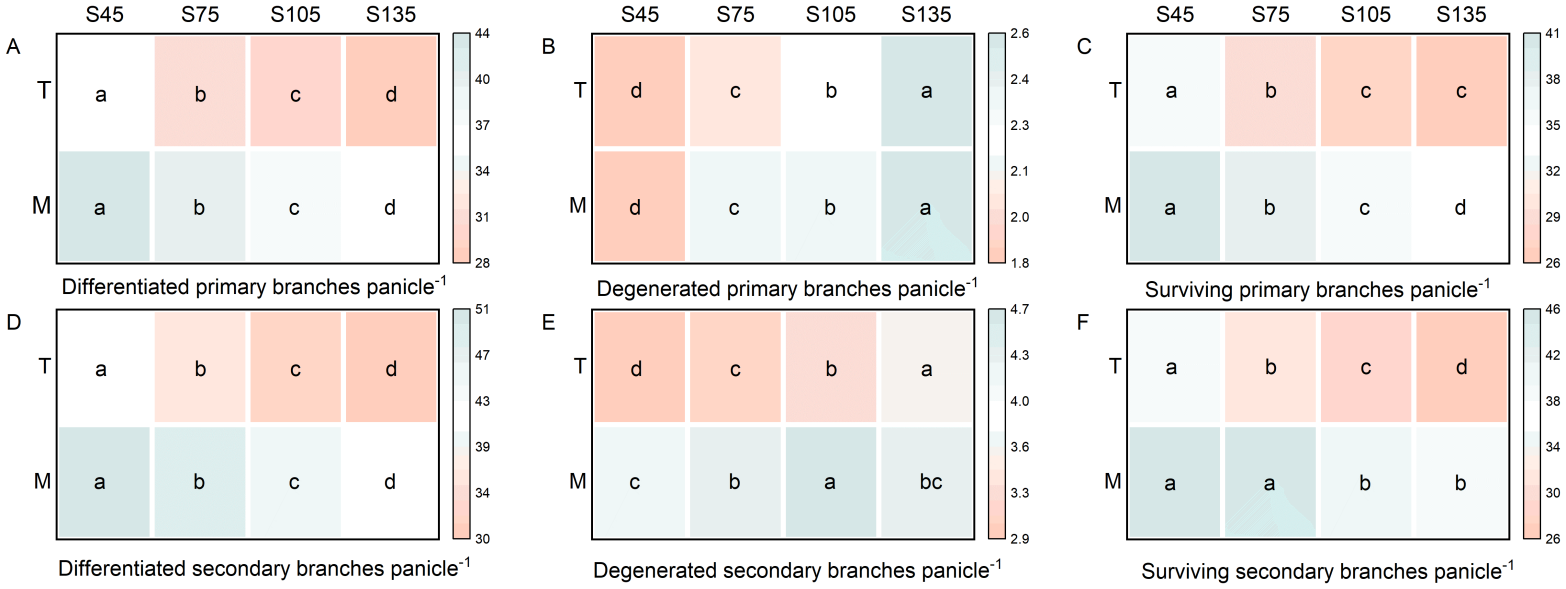
Effects of different seeding rates on the differentiation and degeneration of primary and secondary branch spikelets in direct-seeded rice in 2023 year. S45: 45 kg hm^-2^; S75: 75 kg hm^-2^; S105: 105 kg hm^-2^; S135: 135 kg hm^-2^. **(A–C)** primary branches; **(D–F)** secondary branches. M, main stem; T, tiller. Different letters indicate significant differences between treatments (*P* < 0.05).

As the seeding rate increased from S45 to S135, the number of differentiated and surviving primary and secondary branch spikelets per panicle for both main stems and tillers in direct-seeded rice followed the order of S45 > S75 > S105 > S135 (**Figures 3A, C, D, F**). The number of degenerated spikelets exhibited the reverse trend, following the order of S135 > S105 > S75 > S45 (**Figures 3B, E**). Under identical seeding rates, the main stem demonstrated a higher number of differentiated and surviving primary and secondary branch spikelets per panicle compared to tillers, while the number of degenerated spikelets was lower in the main stem than in tillers (**Figure 3**).

### 3.4 Effects of different seeding rates on the 1000-grain weight and seed setting rate of primary and secondary branches in direct-seeded rice

**Figure 4:**
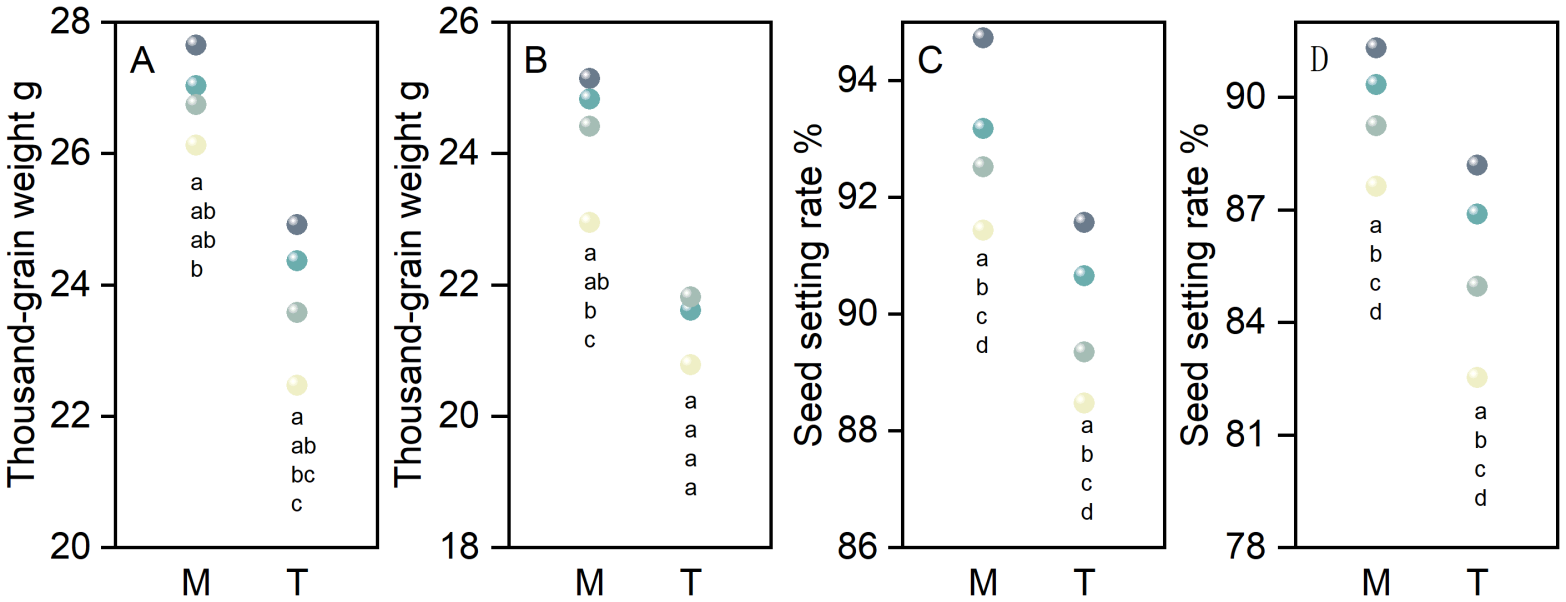
Effects of different seeding rates on the 1000-grain weight and seed setting rate of primary and secondary branches in direct-seeded rice in 2023 year. S45: 45 kg hm^-2^; S75: 75 kg hm^-2^; S105: 105 kg hm^-2^; S135: 135 kg hm^-2^. **(A, C)** primary branches; **(B, D)** secondary branches. M, main stem; T, tiller. Different letters indicate significant differences between treatments (*P* < 0.05).

As the seeding rate increased from S45 to S135, the 1000-grain weight of primary and secondary branches for both main stems and tillers in direct-seeded rice exhibited a decreasing trend, following the order of S45 > S75 > S105 > S135 (**Figures 4A, B**). Similarly, the seed setting rate of primary and secondary branches for both main stems and tillers decreased progressively with increasing seeding rates, reaching its highest value at S45 (**Figures 4C, D**). Under identical seeding rates, the 1000-grain weight and seed setting rate of primary and secondary branches were consistently higher in main stems compared to tillers in direct-seeded rice (**Figure 4**).

### 3.5 Effects of different seeding rates on stem morphological characteristics of direct-seeded rice

**Figure 5:**
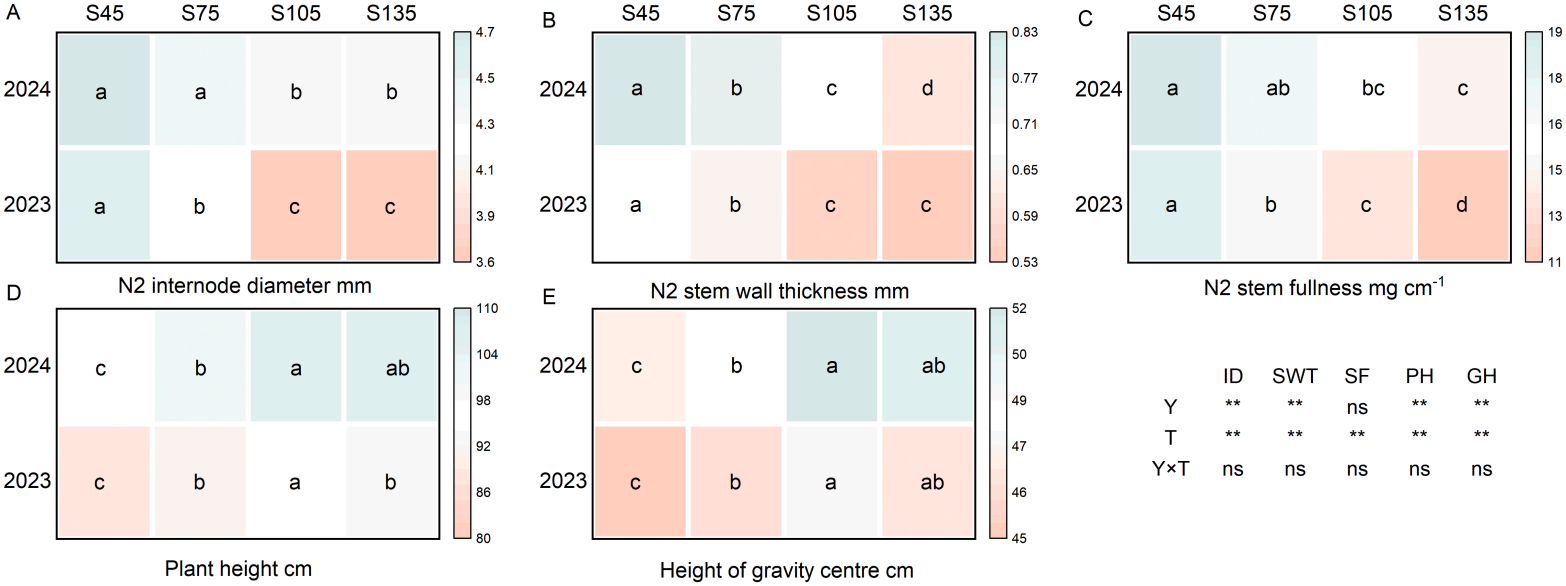
Effects of different seeding rates on stem morphological characteristics of direct-seeded rice in 2023 and 2024 year. S45: 45 kg hm^-2^; S75: 75 kg hm^-2^; S105: 105 kg hm^-2^; S135: 135 kg hm^-2^. **(A)** N2 internode diameter; **(B)** N2 stem wall thickness; **(C)** N2 stem fullness; **(D)** plant height; **(E)** height of gravity center. ID, N2 internode diameter; SWT, N2 stem wall thickness; SF, N2 stem fullness; PH, plant height; GH, height of gravity center; Y, year; T, treatment. Different letters indicate significant differences between treatments (*P* < 0.05). **Significant at *p*<0.01; ns significant at *p*>0.05.

Year and seeding rate exerted a significant influence on the plant height, height of the center of gravity, second internode diameter, and stem wall thickness of direct-seeded rice. Conversely, the interaction between year and seeding rate did not have a notable impact on the stem morphological characteristics of direct-seeded rice (**Figure 5**). As the seeding rate increased from S45 to S135, the diameter of the second internode, stem wall thickness, and stem fullness showed a consistent decreasing trend, following the order of S45 > S75 > S105 > S135 (**Figures 5A–C**). Additionally, both the plant height and the height of the center of gravity in direct-seeded rice exhibited an initial increase followed by a decrease, with their maximum values occurring at S105 (**Figures 5D, E**).

### 3.6 Effects of different seeding rates on mechanical properties of stem in direct-seeded rice

**Figure 6:**
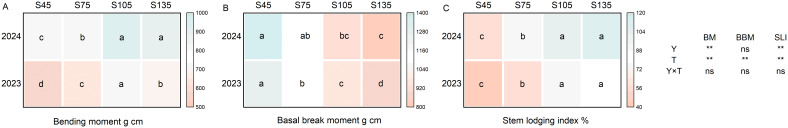
Effects of different seeding rates on mechanical properties of stem in direct-seeded rice in 2023 and 2024 year. S45: 45 kg hm^-2^; S75: 75 kg hm^-2^; S105: 105 kg hm^-2^; S135: 135 kg hm^-2^. **(A)** bending moment; **(B)** basal break moment; **(C)** stem lodging index. BM, bending moment; BBM, basal break moment; SLI, stem lodging index; Y, year; T, treatment. Different letters indicate significant differences between treatments (*P* < 0.05). **Significant at *p*<0.01; ns significant at *p*>0.05.

Year and seeding rate exerted a significant influence on the bending moment and stem lodging index of direct-seeded rice. Conversely, the interaction between year and seeding rate did not have a notable impact on the mechanical properties of stem of direct-seeded rice (**Figure 6**). As the seeding rate increased from S45 to S135, the bending moment of direct-seeded rice stems followed the order of S105 > S135 > S75 > S45 (**Figure 6A**). The basal break moment exhibited a decreasing trend, following the order of S45 > S75 > S105 > S135 (**Figure 6B**). Additionally, the stem lodging index demonstrated an increasing trend with higher seeding rates, following the order of S135 > S105 > S75 > S45 (**Figure 6C**).

### 3.7 Effects of different seeding rates on the physicochemical components of direct-seeded rice stems

**Figure 7:**
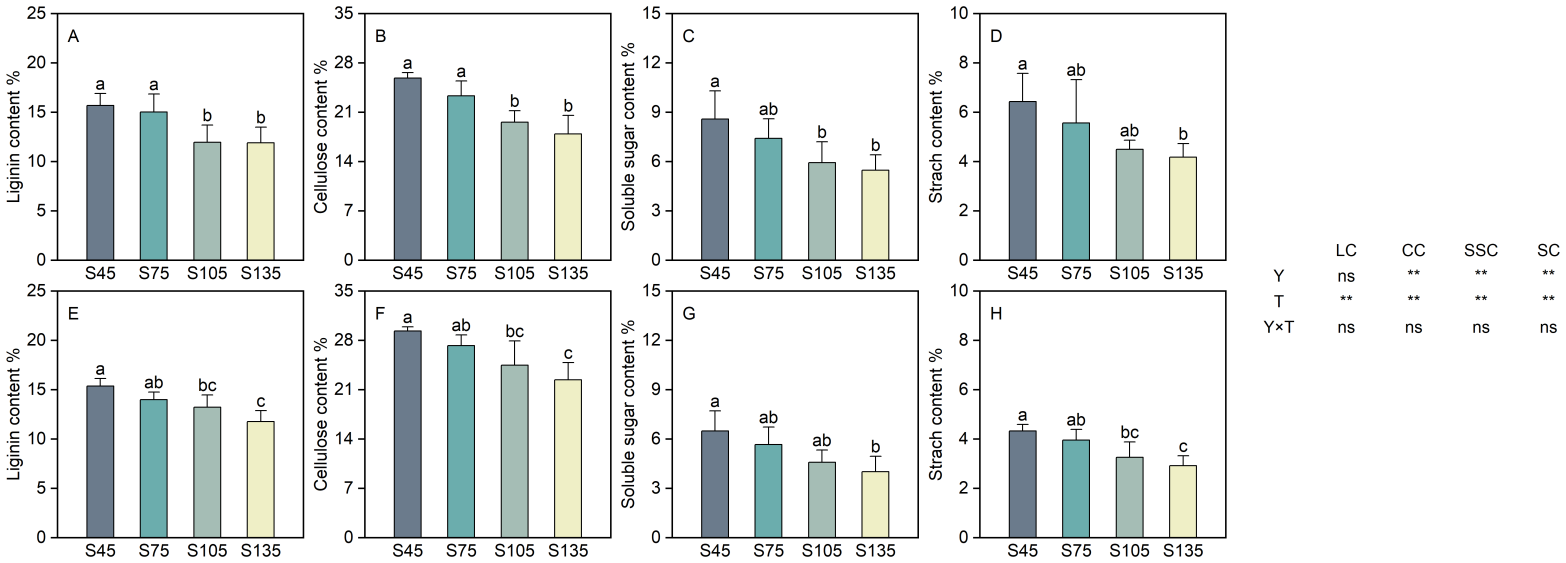
Effects of different seeding rates on the physicochemical components of direct-seeded rice stems in 2023 and 2024 year. S45: 45 kg hm^-2^; S75: 75 kg hm^-2^; S105: 105 kg hm^-2^; S135: 135 kg hm^-2^. **(A–D)** 2023 year; **(E–H)** 2024 year. Values are means ± SD (n = 3). LC, lignin content; CC, cellulose content; SSC, soluble sugar content; SC, starch content; Y, year; T, treatment. Different letters indicate significant differences between treatments (*P* < 0.05). **Significant at *p*<0.01; ns significant at *p*>0.05.

Year and seeding rate exerted a significant influence on the cellulose, soluble sugar, and starch contents of direct-seeded rice. Conversely, the interaction between year and seeding rate did not have a notable impact on the physicochemical components of direct-seeded rice (**Figure 7**). As the seeding rate increased from S45 to S135, the lignin, cellulose, soluble sugar, and starch contents in direct-seeded rice stems exhibited a consistent decreasing trend, following the order of S45 > S75 > S105 > S135 (**Figure 7**). Compared with S45, the lignin content decreased by 6.60%–23.73% (two-year average) from S75 to S135; the cellulose content decreased by 8.37%–27.09% (two-year average); the soluble sugar content decreased by 13.27%–37.29% (two-year average); and the starch content decreased by 10.96%–33.78% (two-year average).

### 3.8 Effects of different seeding rates on root morphological traits and root lodging index of direct-seeded rice

**Figure 8:**
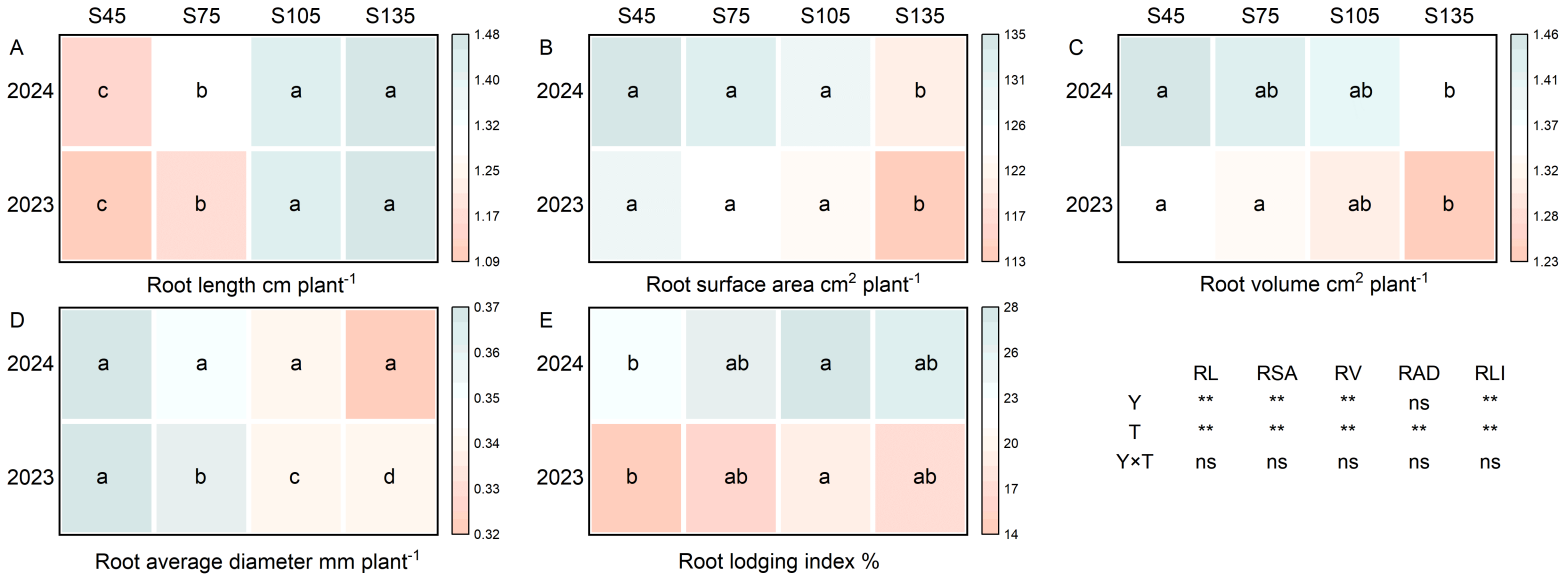
Effects of different seeding rates on root morphological traits and root lodging index of direct-seeded rice in 2023 and 2024 year. S45: 45 kg hm^-2^; S75: 75 kg hm^-2^; S105: 105 kg hm^-2^; S135: 135 kg hm^-2^. **(A)** root length; **(B)** root surface area; **(C)** root volume; **(D)** root average diameter; **(E)** root lodging index. RL, root length; RSA, root surface area; RV, root volume; RAD, root average diameter; RLI, root lodging index; Y, year; T, treatment. Different letters indicate significant differences between treatments (*P* < 0.05). **Significant at *p*<0.01; ns significant at *p*>0.05.

Year and seeding rate exerted a significant influence on the root length, root surface area, root volume, and root lodging index of direct-seeded rice. Conversely, the interaction between year and seeding rate did not have a notable impact on the root morphological traits and root lodging index of direct-seeded rice (**Figure 8**). As the seeding rate increased from S45 to S135, the root length of direct-seeded rice exhibited an increasing trend, following the order of S135 > S105 > S75 > S45 (**Figure 8A**). In contrast, the root average diameter, root volume, and root surface area demonstrated a decreasing trend, following the order of S45 > S75 > S105 > S135 (**Figures 8B–D**). Compared with S45, the root lodging index for S75 to S135 increased by an average of 12.15%–26.12% over the two years (**Figure 8E**).

### 3.9 Correlation analysis and grey relational analysis

**Figure 9:**
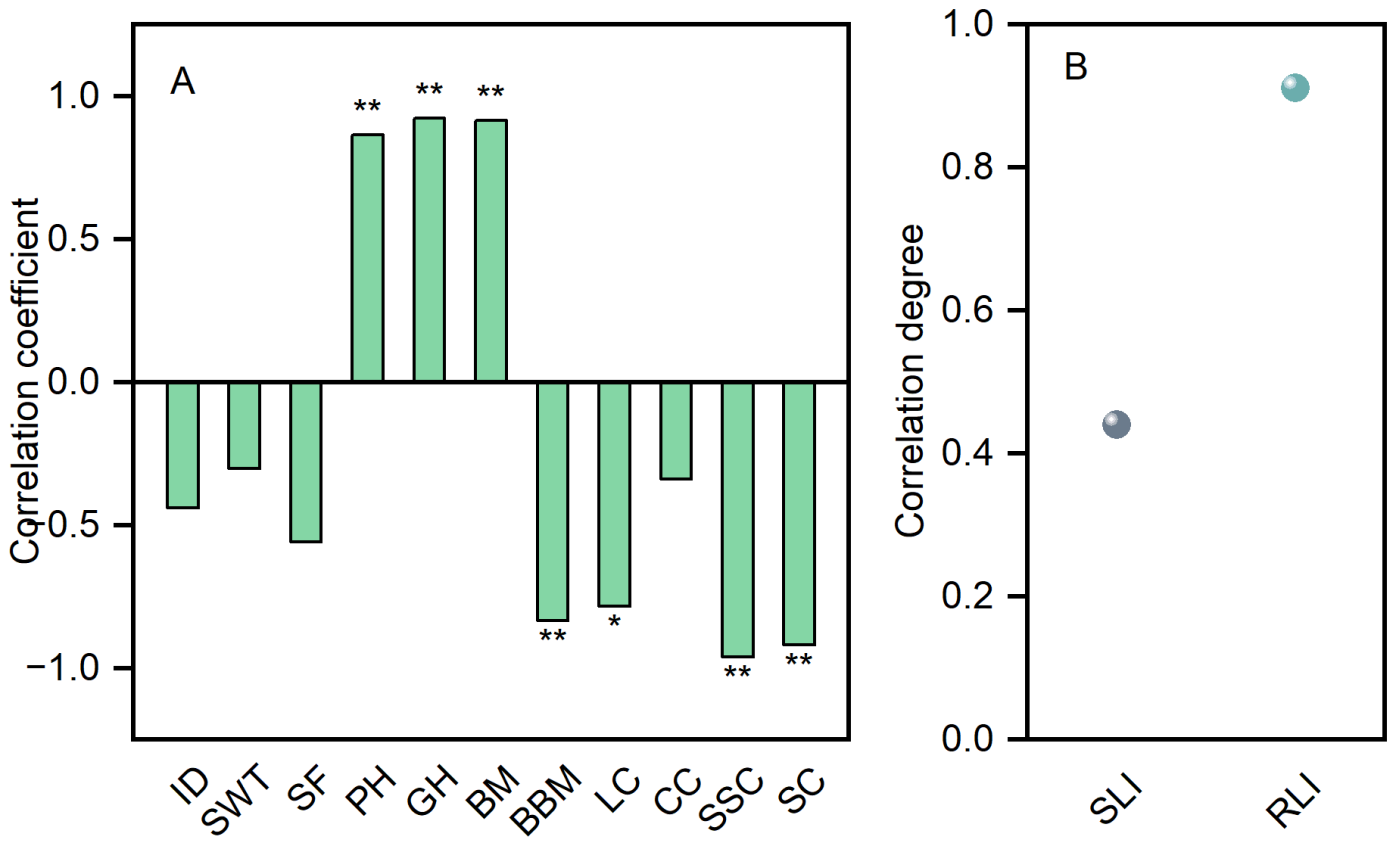
Correlation analysis **(A)** and grey relational analysis **(B)**. ID, N2 internode diameter; SWT, N2 stem wall thickness; SF, N2 stem fullness; PH, plant height; GH, height of gravity center; BM, bending moment; BBM, basal break moment; LC, lignin content; CC, cellulose content; SSC, soluble sugar content; SC, starch content; SLI, stem lodging index; RLI, root lodging index. *Significant at *p*<0.05; **Significant at *p*<0.01.

The lodging resistance index of direct-seeded rice stems is negatively correlated with the diameter of the second internode, the thickness of the second internode wall, the fullness of the second internode, the lignin content of the second internode, the cellulose content of the second internode, the soluble sugar content of the second internode, the starch content of the second internode, and the basal break moment. Conversely, the lodging resistance index is positively correlated with plant height, center of gravity height, and bending moment (**Figure 9A**). Additionally, the root lodging index demonstrated a higher correlation with yield compared to the stem lodging index (**Figure 9B**).

### 3.10 Effects of different seeding rates on direct-seeded rice yield

**Figure 10:**
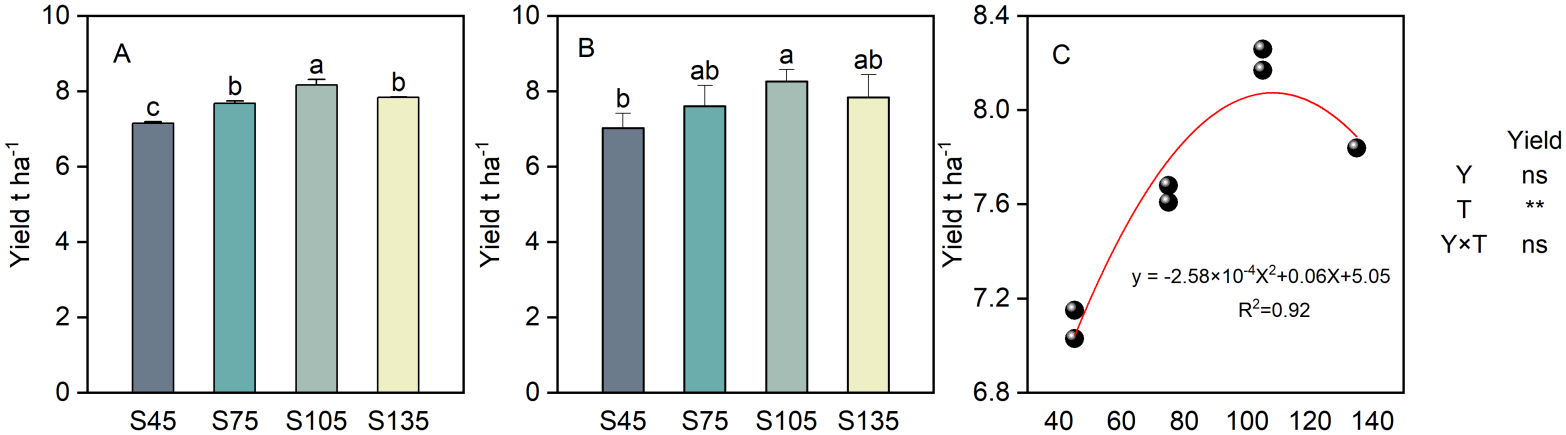
Effects of different seeding rates on direct-seeded rice yield in 2023 and 2024 year. S45: 45 kg hm^-2^; S75: 75 kg hm^-2^; S105: 105 kg hm^-2^; S135: 135 kg hm^-2^. **(A)** 2023 year; **(B)** 2024 year; **(C)** regression analysis. Values are means ± SD (n = 3). Y, year; T, treatment. Different letters indicate significant differences between treatments (*P* < 0.05). **Significant at *p*<0.01; ns significant at *p*>0.05.

The interaction between year and seeding rate did not have a notable impact on the yield of direct-seeded rice (**Figure 10**). As the seeding rate increased from S45 to S135, the yield of direct-seeded rice followed the order: S105 > S135 > S75 > S45 (**Figures 10A, B**). A regression analysis was performed between the direct-seeded rice yield (y) and the seeding rate (x) (**Figure 10C**), yielding a quadratic equation: y = -2.58x² + 0.06x + 5.05, with the optimal seeding rate (x) being 108 kg ha^-1^. Therefore, the optimal seeding rate for achieving high yield in direct-seeded rice is within the range of 105 kg ha^-1^ to 108 kg ha^-1^. When the seeding rate exceeds 108 kg ha^-1^, the yield exhibits a downward trend.

## 4 Discussion

### 4.1 Effects of different seeding rates on tillering of direct-seeded rice

Rice tillering can be categorized into effective tillering and ineffective tillering, with effective tillering serving as the foundation for yield formation. In agricultural production, efforts are focused on enhancing effective tillering while reducing ineffective tillering to improve productive tillers and thereby increase rice yield. The productive tillers is determined by the maximum number of tillers, the number of effective panicles, and the tillering ability of individual plants. Enhancing the tillering capacity of individual plants and controlling the occurrence of ineffective tillers are critical strategies for improving the productive tillers of rice (Rokonuzzaman et al., 2011; Guo et al., 2023; Liu et al., 2024). In this study, as the seeding rate increased, the productive tillers and individual tillering ability of direct-seeded rice followed the order: S45 > S75 > S105 > S135; the number of effective panicles and the maximum number of tillers followed the order: S135 > S105 > S75 > S45 (**Figure 1**). This indicates that when the seeding rate is excessively high, intra-population competition intensifies, leading to a reduction in the tillering ability of individual plants in direct-seeded rice. Simultaneously, more tillers are converted into ineffective tillers, which cannot develop into effective panicles, ultimately resulting in a decline in the productive tillers. Additionally, in this study, as the seeding rate increased, the yield of direct-seeded rice followed the order: S105 > S135 > S75 > S45 (**Figure 10**). This suggests that at low seeding rates, the tillering ability of individual plants is enhanced, but the number of effective panicles is relatively small, making it challenging to achieve high yields. Conversely, at high seeding rates, the contradiction between individual plants and the population intensifies, hindering the establishment of a reasonable population structure and leading to a decrease in the yield of direct-seeded rice.

### 4.2 Effects of different seeding rates on heterogeneity of main stems and tillers in direct-seeded rice

Main stems and tillers serve as critical foundations for constructing a reasonable population structure. The yield components of main stems and tillers contribute differently to overall yield. Increasing the number of spikelets per panicle, seed setting rate, and 1000-grain weight of tillers is one of the important strategies for enhancing crop yield (Counce et al., 1996; Maidl et al., 2010; Arduini et al., 2018; Yang et al., 2022). The number of spikelets per panicle is a key factor determining yield in high-yield cultivation (Lu et al., 2022). It represents the culmination of physiological processes such as spikelet differentiation and degeneration in rice (Liu et al., 2022). The growth and development of rice spikelets are influenced by variety characteristics, cultivation practices, and climatic conditions (Yao et al., 2000; Liu et al., 2022; Zhang et al., 2022). In this study, as the seeding rate increased, the number of differentiated and surviving spikelets of the primary and secondary branches of both main stems and tillers in direct-seeded rice gradually decreased, reaching the lowest at S135; the number of degenerated spikelets increased, reaching the highest at S135 (**Figure 3**). This indicates that a high seeding rate limits the differentiation ability of the primary and secondary branches of spikelets in direct-seeded rice and exacerbates spikelet degeneration, thereby reducing the number of spikelets per panicle in direct-seeded rice. Under the same seeding rate, the number of differentiated and surviving spikelets of the primary and secondary branches of main stems was higher than that of tillers, and the degeneration rate was lower (**Figure 3**). This is one of the reasons why the panicle weight of main stems exceeds that of tillers. Individual plants determine the yield and quality of the population. Differences in the growth and development of main stems and tillers result in varying levels of productivity. Chlorophyll is a crucial substance that determines photosynthetic strength and serves as the basis for dry matter accumulation (Wang et al., 2012). In this study, as the seeding rate increased, the chlorophyll content of both main stems and tillers gradually decreased, reaching the lowest at S135; under the same seeding rate, the chlorophyll content of main stems was higher than that of tillers (**Figure 2**). This indicates that a low seeding rate promotes the production of more photosynthetic products in direct-seeded rice, enhancing the seed setting rate and 1000-grain weight (**Figure 4**). Meanwhile, the seed setting rate and 1000-grain weight of main stem panicles were greater than those of tiller panicles, which may be related to photosynthetic material production, assimilate transport, and assimilate utilization during grain development in the later stages. An increase in seeding rate reduces the number of spikelets per panicle, seed setting rate, and 1000-grain weight of individual plants but increases population yield. The possible reason is that an increase in seeding rate raises the number of effective panicles per unit area, and the advantage of population quantity compensates for the deficiency in individual productivity (**Figures 1**, **2**, **4**, **10**). Additionally, in this study, the total leaf area of main stems in direct-seeded rice exhibited an initial increase followed by a decrease with increasing seeding rate, reaching the maximum at S105; the leaf area of tillers gradually decreased, reaching the minimum at S45 (**Figure 2**). This indicates that an appropriate seeding rate ensures the number of main stems in direct-seeded rice, increases leaf area, promotes photosynthetic material accumulation, and enhances yield. However, a high seeding rate causes severe mutual shading among leaves in the direct-seeded rice population, reduces the light-receiving area of individual leaves, and intensifies competition between main stems and tillers, leading to a reduction in the leaf area of tillers.

### 4.3 Effects of seeding rate on lodging of direct-seeded rice

Lodging is one of the critical factors affecting the yield potential of rice (Hong et al., 2022). The lodging index is commonly regarded as an indicator of rice lodging resistance, with a higher lodging index indicating a greater risk of lodging (Chen et al., 2021; Wu et al., 2024). In this study, the lodging index of direct-seeded rice stems gradually increased with increasing seeding rate and reached its maximum at S135. The possible reason is that a significant increase in seeding rate enhances the bending moment while significantly reducing the basal break strength of base second internode, thereby increasing the lodging index of direct-seeded rice stems. Although the bending moment was the largest at S105, the decrease in basal break strength at S135 was greater than the increase in bending moment, resulting in a higher lodging index at S135 compared to S105. This indicates that the basal break strength is the key determinant of stem lodging resistance.

Plant height and center of gravity height are important phenotypic traits influencing crop lodging resistance (Ahmad et al., 2023). In this study, as the seeding rate increased, the plant height and center of gravity height of direct-seeded rice followed the order: S105 > S135 > S75 > S45 (**Figure 5**). The possible reason is that an increase in seeding rate causes leaf overlap and shading, prompting plants to rapidly elongate their stems to adapt to shading stress, thereby increasing plant height and center of gravity height in direct-seeded rice (Hisae and Kouki, 2011). However, at the high seeding rate of S135, individual plant development deteriorates, leading to shorter plants and reduced plant height and center of gravity height. Plant height and center of gravity height are significantly positively correlated with the lodging rate of crops, and the greater these heights, the stronger the negative impact on crop lodging resistance (Navabi et al., 2006; Liu et al., 2024). In this study, the lodging index of direct-seeded rice stems was significantly positively correlated with plant height and center of gravity height (**Figure 9**). Therefore, an appropriate seeding rate is beneficial for shortening internode elongation, inhibiting plant height growth, reducing the lodging index, and thereby enhancing the lodging resistance of direct-seeded rice.

The base second internode of the rice stem is the critical part where lodging occurs (Zhang et al., 2017; Jiang et al., 2025). The stem diameter and wall thickness of rice are significantly positively correlated with stem breaking strength, and increasing stem diameter and wall thickness can significantly enhance stem lodging resistance (Lu et al., 2025; Xu et al., 2024). In this study, as the seeding rate increased, the diameter, wall thickness, and fullness of base second internode of direct-seeded rice all decreased, reaching their maximum at S45; the diameter, wall thickness, and fullness of the second internode were negatively correlated with the lodging index (**Figures 5**, **9**). These results indicate that at high seeding rates, the wall thickness of the stem becomes thinner, hardness decreases, and unit length stem weight reduces. At low seeding rates, it is advantageous for thickening the stem and stem wall of the second internode and increasing internode fullness. Simultaneously, the contents of lignin, cellulose, starch, and soluble sugar in the second internode decreased with increasing seeding rate and were positively correlated with the lodging index (**Figures 7**, **9**). This suggests that at high seeding rates, the reduction in various physical and chemical components regulates the mechanical toughness of the second internode, thereby decreasing the lodging resistance of direct-seeded rice. At low seeding rates, the smaller population density allows individual plants to increase the contents of starch, soluble sugar, lignin, and cellulose in the second internode to strengthen the cell wall, increase internode fullness and breaking strength, and reduce the risk of lodging.

Whether the root system develops well is the foundation for achieving high yield. A developed and vigorous root system has a stronger ability to absorb water and nutrients from the soil and provides more nutrients to the aboveground parts. An excellent root system is characterized by deep roots, large volume, appropriate spatial distribution, and active root activity, enabling the coexistence of high yield and lodging resistance in crops (Gao et al., 2022; Liang et al., 2025; Sun et al., 2022). In this study, as the seeding rate increased, the root length of direct-seeded rice gradually increased, reaching its maximum at S135; however, the root diameter, root volume, and root surface area gradually decreased, reaching their minimum at S135 (**Figure 8**). This indicates that an excessive seeding rate intensifies the contradiction between root growth and aboveground development, weakens root system nutrient output, and leads to a decrease in the number of spikelets per panicle, 1000-grain weight, and seed setting rate with increasing seeding rate. Eventually, although the seeding rate of S135 was higher than that of S105, the yield of S135 was lower than that of S105. At the same time, due to the weakening of root system growth caused by high seeding rates, aboveground stem development was poor, stem bending resistance and toughness decreased, and lodging resistance weakened, increasing the root lodging index (**Figure 8**). The correlation between the root lodging index and yield was higher than that between the stem lodging index and yield (**Figure 9**). Therefore, in research aimed at reducing the lodging risk of direct-seeded rice, attention should be paid to both stem lodging and root lodging.

### 4.4 Effects of seeding rate on direct-seeded rice yield

Rice yield is determined by four key factors: the number of effective panicles, the number of spikelets per panicle, the seed setting rate, and the 1000-grain weight. Optimizing cultivation practices to coordinate the interactions among these yield components can comprehensively enhance rice yield. In this study, the number of effective panicles in direct-seeded rice increased with increasing seeding rate, reaching its maximum at the S135. Conversely, the number of spikelets per panicle, the seed setting rate, and the 1000-grain weight decreased with increasing seeding rate, reaching their maximum values at the S45. The yield of direct-seeded rice exhibited an initial increase followed by a decrease with increasing seeding rate, peaking at the S105(**Figures 1**, **3**, **4**, **10**). This indicates that a high number of effective panicles does not necessarily lead to high yield, and a high number of spikelets per panicle, seed setting rate, and 1000-grain weight do not necessarily result in the highest yield. Yield is determined by the combined effects of all yield components. Meanwhile, in this study, the optimal seeding rate for direct-seeded rice was determined to be 105 kg ha^-1^ to 108 kg ha^-1^(**Figure 10**). Within a certain range, increasing the seeding rate has relatively little impact on the contradiction between individual plants and the population in direct-seeded rice. The optimal seeding rate ensures good individual development while increasing the number of effective panicles per unit area of the direct-seeded rice population, which is conducive to enhancing yield. However, an excessively high seeding rate intensifies the contradiction between individual plants and the population. The direct-seeded rice population becomes too dense, hindering individual growth and affecting the quality of individual development. This not only reduces stem lodging resistance but also gradually decreases the 1000-grain weight, spikelets number, and seed setting rate. The accumulation of grain material per plant decreases, leading to a reduction in overall grain material accumulation and ultimately impacting population yield. The S105 seeding rate not only promotes the development of individual plants but also achieves the best synergy among all yield components, resulting in the highest yield.

## 5 Conclusion

An appropriate seeding rate for direct-seeded rice can not only construct a rational population structure and ensure an adequate number of effective panicles but also optimize individual plant development, enhance lodging resistance and photosynthetic efficiency, thereby contributing to increased yield in direct-seeded rice.

## Data Availability

The raw data supporting the conclusions of this article will be made available by the authors, without undue reservation.
